# Survival outcomes following craniotomy for intracranial metastases from an unknown primary

**DOI:** 10.1007/s10147-020-01687-w

**Published:** 2020-05-01

**Authors:** Melissa Gough, Molly Nielsen, Ian C. Coulter, Damian Holliman

**Affiliations:** 1 School of Medical Education, Newcastle University Medical School, The Medical School, Newcastle University, Framlington Place, Newcastle upon Tyne, NE2 4HH UK; 2 Department of Neurosurgery, Royal Victoria Infirmary, Newcastle upon Tyne, UK

**Keywords:** Intracranial, Metastasis, Primary, Survival, Unknown

## Abstract

**Introduction:**

Management of patients with intracranial metastases from an unknown primary tumor (CUP) varies compared to those with metastases of known primary tumor origin (CKP). The National Institute for Health and Care Excellence (NICE) recognizes the current lack of research to support the management of CUP patients with brain metastases. The primary aim was to compare survival outcomes of CKP and CUP patients undergoing early resection of intracranial metastases to understand the efficacy of surgery for patients with CUP.

**Methods:**

A retrospective study was performed, wherein patients were identified using a pathology database. Data was collected from patient notes and trust information services. Surgically managed patients during a 10-year period aged over 18 years, with a histological diagnosis of intracranial metastasis, were included.

**Results:**

298 patients were identified, including 243 (82.0%) CKP patients and 55 (18.0%) CUP patients. Median survival for CKP patients was 9 months (95%CI 7.475–10.525); and 6 months for CUP patients (95%CI 4.263–7.737, *p* = 0.113). Cox regression analyses suggest absence of other metastases (*p* = 0.016), age (*p* = 0.005), and performance status (*p* = 0.001) were positive prognostic factors for improved survival in cases of CUP. The eventual determination of the primary malignancy did not affect overall survival for CUP patients.

**Conclusions:**

There was no significant difference in overall survival between the two groups. Surgical management of patients with CUP brain metastases is an appropriate treatment option. Current diagnostic pathways specifying a thorough search for the primary tumor pre-operatively may not improve patient outcomes.

## Keypoints

1. NICE guidance does not detail specific management for CUP patients with intracranial metastases.
2. CUP patients with intracranial metastases and those with known primary (CKP) were compared.
3. No significant difference in survival between CUP and CKP groups after surgery for metastases.

## Importance of the study

Patients with cancer of an unknown primary (CUP) comprise around 5 – 15% of total cerebral metastases cases, according to a range of sources. NICE guideline, *Brain tumours (primary) and brain metastases in adults *(NG99, 2018) does not detail specific management guidance for CUP patients with intracranial metastases. The findings within this study support data elsewhere in the literature that survival outcomes after early resection of intracranial metastases in CUP patients are not significantly different from those in CKP patients. Cox regression analysis within this study revealed that single metastasis, younger age and better Eastern Cooperative Oncology Group (ECOG) performance status are associated with better outcomes in CUP patients undergoing resection of intracranial metastases. We advocate large-scale prospective studies of this patient group to further refine the best treatment options.

## Introduction

Patients with cancer of an unknown primary (CUP) comprise approximately 5–15% of total cerebral metastases cases [8, 22, 24, 26, 33]. Patients presenting with intracranial metastases from a CUP may not be considered for the same surgical treatment options as those afforded to patients with a known primary cancer (CKP) as knowledge of the primary disease’s prognosis typically facilitates treatment decisions [2]. The approach of reserving surgery until a primary is diagnosed may negate the potential benefits of early surgical management, as is more readily offered to those patients with CKP metastases.

In the United Kingdom in 2010, the National Institute for Health and Care Excellence (NICE) recognized a lack of national research strategy to address the needs of CUP patients with brain metastases [19]. In the NICE Managing metastatic malignant disease of unknown primary origin in adults: diagnosis and management pathways guidance (2018) [21], however, it is recommended that patients and carers be informed that there is no evidence that any treatment offers improved survival nor does surgery improve neurological symptoms in those with multiple brain metastases [21].

NICE has previously noted a lack of adequate epidemiological data regarding these patients [19]. In the cases of solitary metastases from a CKP a referral to the neuro-oncology multi-disciplinary team (MDT) is advised for consideration of radical therapy including surgical excision. There is no specific guidance regarding the management of solitary CUP brain metastases within the updated 2018 NICE guidance (NG99) [20].

We aimed to compare survival of CKP and CUP patients undergoing resection of intracranial metastases to determine whether early neurosurgical intervention is a valid treatment option for patients with CUP.

## Materials and methods

Surgically managed patients aged over 18 years, with a histological diagnosis of intracranial metastasis during the 10-year study period between January 2008 and April 2018, were identified using our institution’s pathology database.

Retrospective data including age, gender, tumor characteristics, mode of adjuvant treatment and Eastern Cooperative Oncology Group Performance Status (ECOG) were collected from patient notes and trust information services using a proforma. The Summary Care Record was interrogated to determine the date of death. Whilst the precise mode of surgical excision was not recorded for this study, surgeons at our centre employ en bloc and piecemeal excision techniques tailored to the particular case. Typically patients receive a post-operative computed tomography (CT) scan to assess for the presence of hematoma and gross residual. An MRI is usually arranged at variable points during follow-up, though this data was not collected and analyzed as part of this study.

Patients were censored from analysis if they were alive at the end of the study period. A diagnosis of CUP was assigned to those patients with no identifiable histological diagnosis regarding primary tumour origin at the time of initial diagnosis of cerebral metastasis.

### Ethical statement

The study was registered with our institution as a retrospective service evaluation quality assurance study. Whilst a study-specific consent form was not required, all patients undergoing surgery had a valid procedural consent form completed which included permission to collect data for service and quality assurance projects.

### Statistics

Multivariate analysis was performed using Cox regression analysis, univariate analysis using the Kaplan–Meier (KM) method with comparisons made using the log rank test and independent t-tests were conducted using IBM SPSS Statistics Version 24. Statistical significance was reported if *p* =  < 0.05.

## Results

Of 326 screened cases, 298 patients met the inclusion criteria (age range 20–83 years; median age 61); this included 267 (82.0%) CKP patients (24 censored (9.0%)) and 59 (18.0%) CUP patients (4 censored (6.8%)). The most common sites of CKP metastases were the cerebellum (30.3%), frontal lobe (18.0%) and parietal lobe (18.0%). The most common CUP metastasis site was the frontal lobe (30.9%), followed by cerebellum (21.8%). Table 1 illustrates the clinical information of the patient cohort.

**Table 1:** Patient demographics and clinical information

Total number of patients	298
Median age at met diagnosis	
*All*	62 years
CKP	60 years (range 20–83)
CUP	64 years (range 43–80)
*Gender ratios*	
All	298
	*N (%)*
Male	113 (37.9)
Female	185 (62.1)
CKP	
Male	93 (38.3)
Female	150 (61.7)
CUP	
Male	21 (38.2)
Female	34 (61.8)
*Tumor location*	
CKP	
Cerebellum	74(30.5)
Frontal	44 (18.1)
Parietal	44 (18.1)
Occipital	31 (12.8)
Temporal	12 (4.9)
Other	38 (15.6)
CUP	
Frontal	17 (30.9)
Cerebellum	12 (21.8)
Parietal	5 (9.1)
Temporal	4 (7.2)
Cerebellopontine angle	3 (5.5)
Occipital	3 (5.5)
Other	11 (20)
*Primary identified pre-operatively (CKP)*	
Site	
Lung	77 (31.7)
Breast	64 (26.3)
Gastrointestinal	25 (10.3)
Melanoma	16 (6.6)
Renal cell	16 (6.6)
Ovarian/endometrial/cervical	14 (5.8)
Prostate	3 (1.2)
Bladder	2 (0.8)
Other	26 (10.7)
*Primary not identified pre-operatively (CUP)*	
Total	55 (38.2)
Identified post-operatively	21 (27.3)
Lung	15 (1.8)
Melanoma	1 (1.8)
Renal	1 (7.2)
Other	4 (61.8)
Never identified	34 (38.2)
*Single versus multiple metastases*	
CKP	
Single	198 (81.5)
Multiple	39 (16.0)
Not recorded	6 (2.5)
CUP	
Single	42 (76.4)
Multiple	11 (20.0)
Not recorded	2 (3.6)
*Post-operative adjuvant therapy*	
CKP	
Radiotherapy	89 (36.6)
Chemotherapy	24 (9.9)
WBRT	14 (5.8)
Radiotherapy and chemotherapy	41 (16.9)
WBRT and chemotherapy	5 (2.0)
SRS	3 (1.2)
SRS and chemotherapy	1 (0.4)
None	60 (24.7)
Not recorded	6 (2.5)
CUP	
Radiotherapy	17 (30.9)
Chemotherapy	5 (9.1)
Radiotherapy and chemotherapy	12 (21.8)
WBRT	3 (5.5)
WBRT and chemotherapy	1 (1.8)
None	13 (23.6)
Not recorded	4 (7.3)

*WBRT* whole brain radiotherapy, *SRS* stereotactic radiosurgery

### Overall survival

**Fig. 1:**
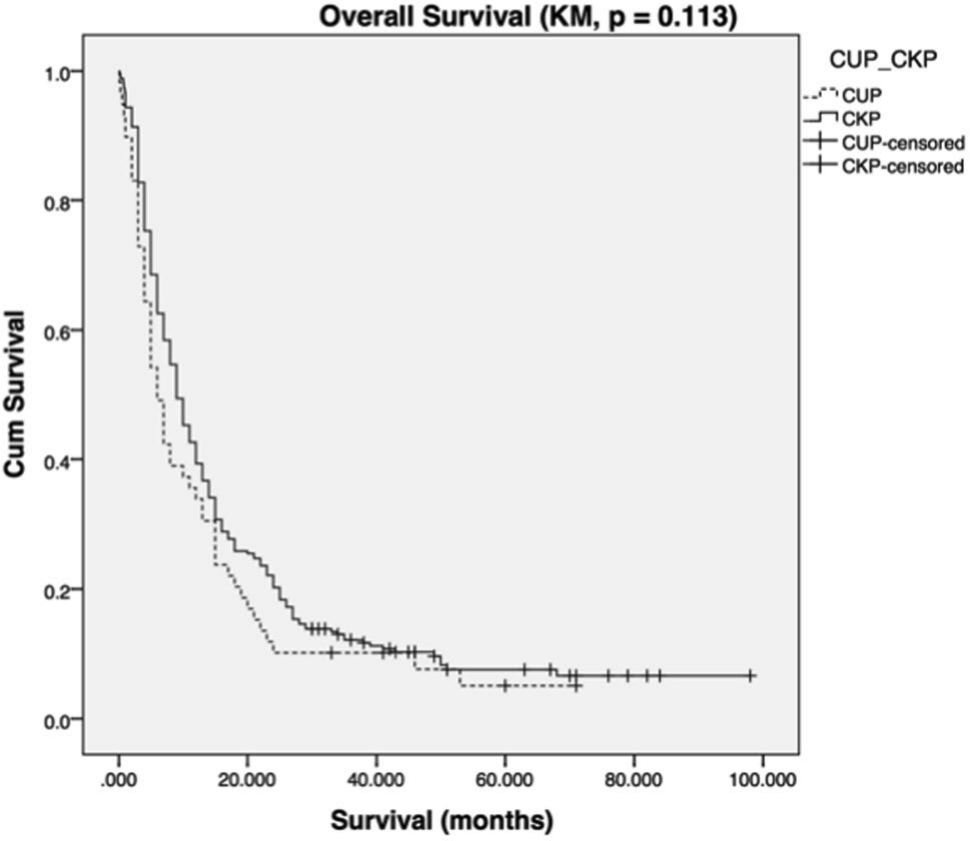
Kaplan–Meier survival plot of CUP versus CKP survival (months)

Univariate analysis revealed a median survival for CKP patients of 9 months (95%CI 7.475–10.525); for CUP patients it was 6 months (95%CI 4.263–7.737, Log rank (Mantel–Cox) *p* = 0.113). Eighty-one patients (33.4%) in the CKP group survived for greater than 1 year after metastasis diagnosis, and a single patient (0.4%) survived 5 years. Sixteen patients (29.1%) from the CUP group survived beyond 1 year, whereas no patients survived to 5 years after metastasis diagnosis (Fig. 1).

### Positive predictive factors

Multivariate Cox regression analysis was performed for both CKP and CUP groups containing sets of six and seven potential prognostic variables, respectively (Table 2).

**Table 2:** CKP and CUP multivariate analysis outputs

Primary tumour	Exp(B)	Lower	Upper	Sig
*CKP*				
Therapy	0.673	0.469	0.965	0.031
Single/multiple	1.652	1.060	2.576	0.027
Recurrence	1.877	1.343	2.623	< 0.0001
Gender	1.036	0.746	1.439	0.833
Other mets	1.260	0.903	1.758	0.173
Age at met diagnosis stratified	1.177	1.004	1.381	0.044
*CUP*				
Therapy	0.177	0.042	0.735	0.017
Single/multiple	0.737	0.233	2.326	0.602
Recurrence	15.750	2.647	93.703	0.002
Gender	1.930	0.505	7.366	0.336
Other mets	9.713	1.533	61.546	0.016
Age at met diagnosis stratified	8.309	1.884	36.647	0.005
Performance status stratified	7.366	2.354	23.050	0.001

### Adjuvant therapy

Adjuvant therapy comprised radiotherapy, chemotherapy, WBRT, SRS or a paired combination (Table 1). There was no statistically significant difference in survival outcome in relation to whether patients received adjuvant monotherapy or combined therapy (*p* = 0.115). Rather, survival outcomes were improved in both CKP (*p* = 0.031) and CUP (*p* = 0.017) groups simply by the addition of any postoperative adjuvant treatment (chemo- and/or radiotherapy).

### 3-Year recurrence

**Fig. 2:**
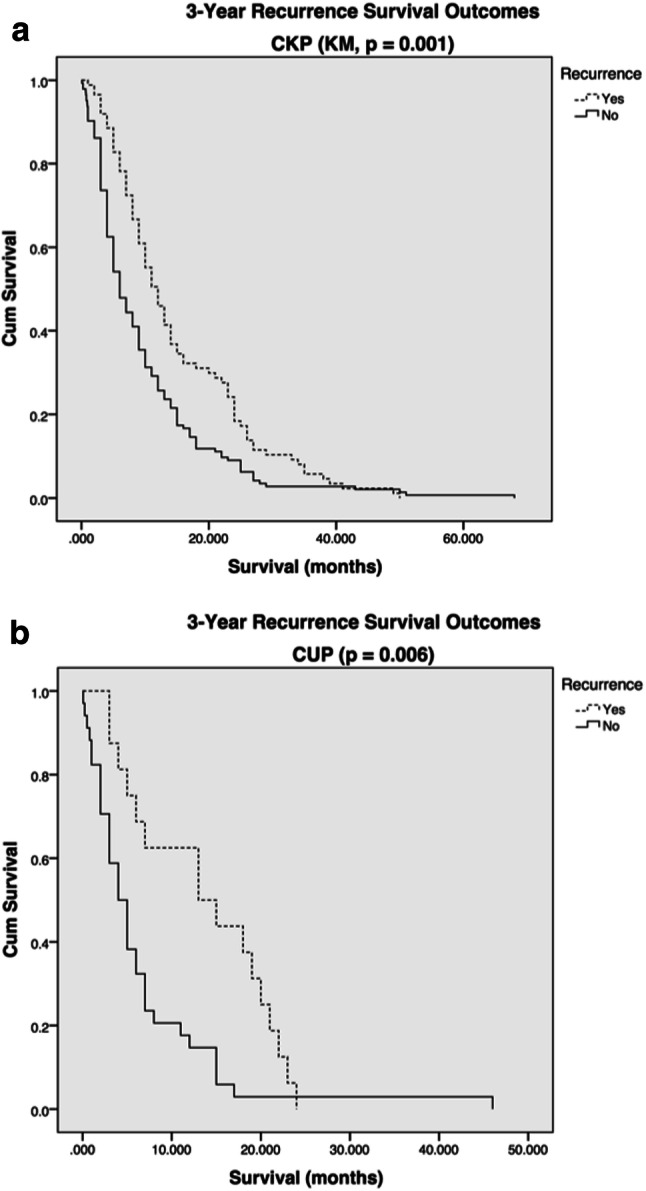
Kaplan–Meier plots representing survival outcome and relationship to incidence of recurrence in **a** CKP and **b** CUP patient groups, survival in months

Median time to recurrence within the CKP group was 7 months (range 0.3–47 months), as opposed to 5 months in the CUP group (range 2–20 months). Patients in the metastasis recurrence group experienced longer survival times than those patients without recurrence. Kaplan–Meier survival analysis of CUP and CKP groups (Fig. 2) demonstrated statistically significant improved survival outcomes among patients with intracranial disease recurrence within both groups CKP (*p* = 0.001) and CUP (*p* = 0.006).

### Survival outcomes stratified by age at metastasis diagnosis

**Fig. 3:**
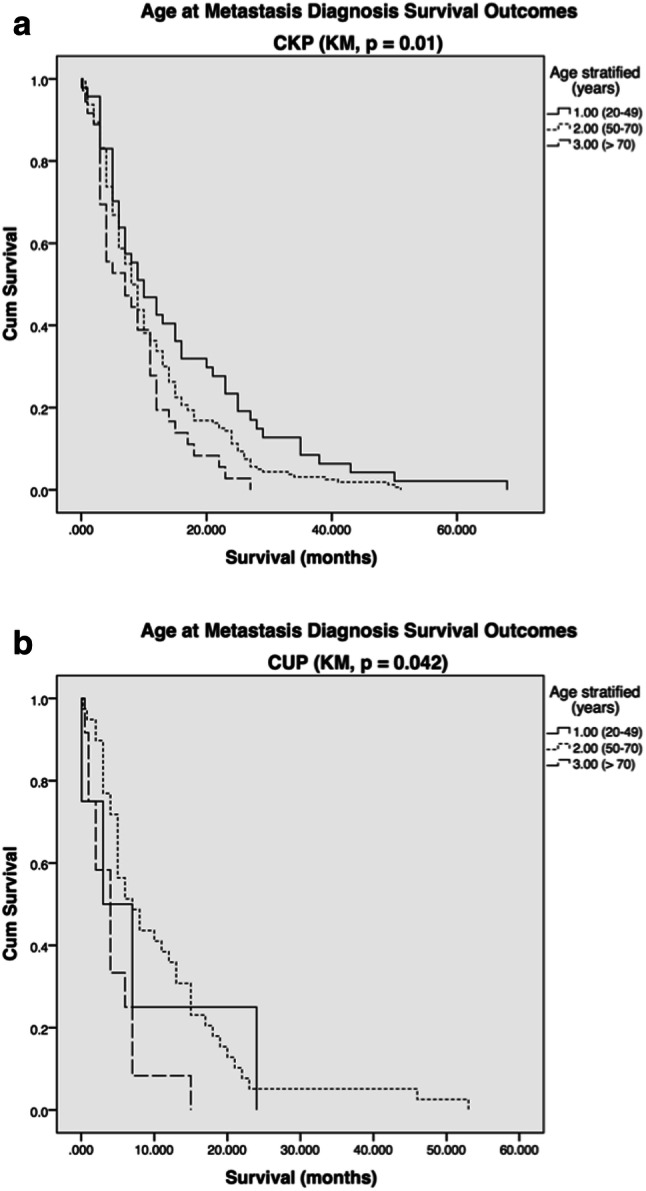
**a** CKP and **b** CUP age at metastasis diagnosis survival outcomes (stratified by age: 1.00 = 20–49; 2.00 = 50–70; 3.00 =  > 71, survival in months)

Survival prognosis deteriorated as patient age increased (*p* = 0.003). This observation was confirmed in analyses of both the CKP (*p* = 0.01) and CUP (*p* = 0.042) patient cohorts (Fig. 3).

### Performance status in CUP patients

**Fig. 4:**
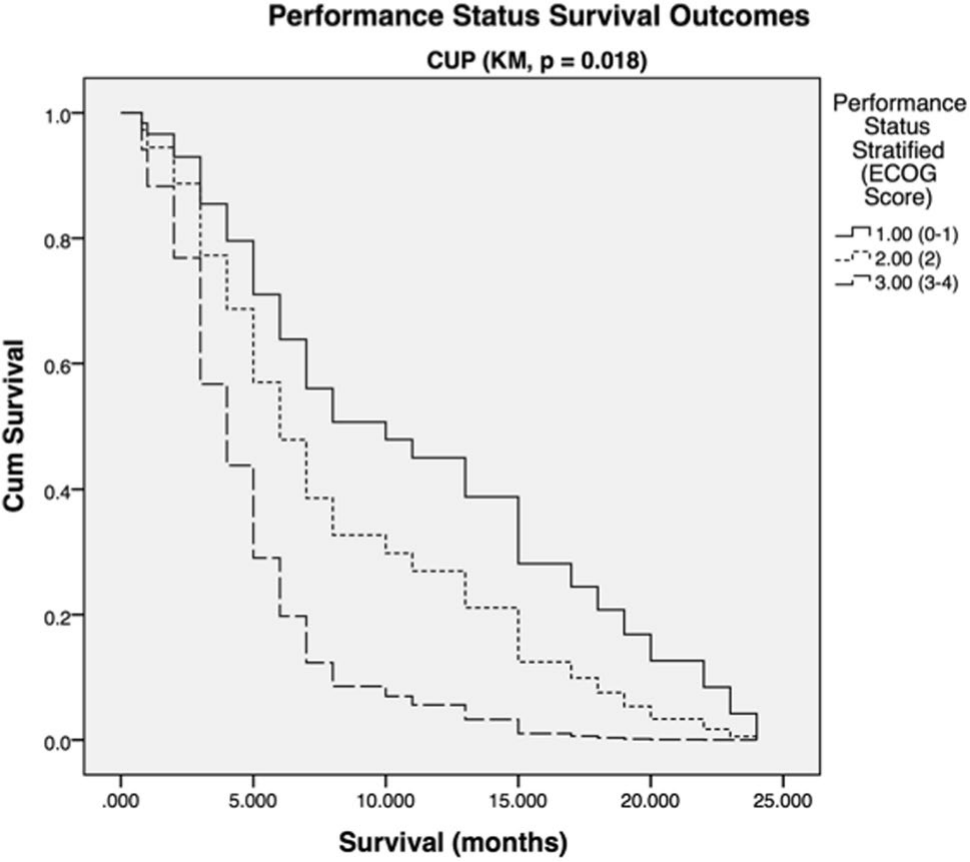
Survival outcome related to ECOG performance score in CUP patient group (1.00 = ECOG 0–1, 2.00 = ECOG 2, 3.00 = ECOG 3–4)

Kaplan–Meier analysis of patients stratified by their performance status revealed that lower Eastern Cooperative Oncology Group (ECOG) performance status significantly correlated with increased survival (*p* = 0.018) (Fig. 4).

### Primary site identification in CUP group

No statistically significant difference in survival outcomes was determined by univariate Kaplan–Meier analysis of CUP patients split into sub-groups according to whether primary site was ever identified (*p* = 0.574).

### Single versus multiple metastases

Both CKP and CUP groups showed preponderance for single cerebral metastasis as opposed to multiple metastases (Table 1). A statistically significant difference was found between CKP patients with single versus multiple metastases (*p* = 0.013), wherein those with a single metastasis experienced longer survival. There was no statistically significant difference in survival outcomes between single or multiple metastasis patients within the CUP group (*p* = 0.581).

### Presence of extracranial metastases

The most common site of extracranial metastases in the CKP group was lymph nodes (*n* = 95, 38.9%), followed by lung (*n* = 62, 25.4%). Forty-eight patients (19.7%) had liver metastases, and 38 (15.6%) had bone metastases. Most patients within the CKP group had other metastases at more than one site. Overall, 163 (66.8%) CKP patients had extra-cranial metastases. Extracranial metastases were found in 26 CUP patients (47.3%). The commonest sites of extra-cranial metastases within the CUP group were lymph nodes and liver (both *n* = 7, 12.7%).

## Discussion

In this retrospective study of 298 patients with brain metastases undergoing surgical excision, there was no significant difference in overall survival between patients with CKP and CUP (9 versus 6 months *p* = 0.113). This study has not identified the reasons for this, but our findings suggest that (a) metastatic disease and (b) cerebral disease and not necessarily the type of cancer, are the major factors that determine a patient’s length of survival.

The median survival for patients with untreated cerebral metastases is 1–2 months, which may be extended to 6 months with surgery, radiotherapy, and chemotherapy [6]. Riihimaki et al. (2013) analyzed a large series comparing 6,745 patients with metastatic CKP at diagnosis and 2,881 patients with metastatic CUP [29]. They observed that CUP patients had poorer survival than patients with CKP, a finding that was not replicated in our cohort. D’Ambrosio and Agazzi found that there was no significant difference in survival between CUP and CKP patients, suggesting that delayed treatment whilst attempting to locate a primary cancer is inappropriate [4]. Whether the primary is eventually found also did not have a statistically significant impact upon survival outcome, an observation replicated in our study [10]. Positive prognostic indicators examined included age < 65 years and treatment modality used [4]. Polyzoidis et al. reached a similar conclusion that identification of the primary tumor does not affect prognosis and survival. Instead, age, Karnofsky Performance Score (KPS), number of metastases, treatment modality, and extent of extracranial pathology [26] were observed to be more favorable prognostic indicators. Unsurprisingly, provision of any postoperative adjuvant therapy has been shown to improve survival outcomes in this series, which is consistent with other studies in the literature [10].

Median survival among CUP patients with brain metastases has been reported to lie anywhere between 4.8–27 months [1, 11, 15, 34]. CUP patients were found to have longer median overall survival than the group containing all known primary brain metastases patients in the series by Bartelt et al. (4.8 and 3.4 months, respectively; *p* = 0.05) [1]. This wide range of outcomes exemplifies the need to obtain further prospective epidemiological data and analysis including stratification of tumor types and treatment modalities as the management of most primary cancers has become varied and complex. Recently Fureder et al. reported no statistical difference in survival between CUP and CKP patients within 3 months of presentation [16] adding further weight to the hypothesis that there is no difference in survival outcomes between CKP and CUP groups undergoing cranial metastasis excision [1, 7, 15, 23, 26]. Interestingly, one study points out that median survival was significantly higher for treated as opposed to untreated CUP patients (3.6 and 1.1 months, respectively; *p* = 0.0001) [10]. This supports the aforementioned conclusions that surgery should not necessarily be deferred for CUP patients if it is deemed potentially efficacious in the initial management phase.

Previous reports have indicated that surgical intervention contributes towards improved survival in CKP brain metastases patients [3, 13, 31, 32]. These demonstrated that neurosurgery coupled with whole brain radiotherapy (WBRT) leads to favorable survival outcomes when compared to the use of WBRT alone (median survival 9–10 months and 3–6 months, respectively) [14] with a lower recurrence rate in those patients undergoing neurosurgical intervention compared to those who did not (20% and 52%, respectively) [18]. Our study has demonstrated that there was no significant difference in survival outcomes between CKP and CUP patient groups following surgical excision. Further research of larger CUP patient populations, via strategies such as national and international databases, is required to elucidate the factors contributing to this finding. Furthermore, we also observed that adjuvant therapy improved survival for patients with CKP and CUP.

Recently ECOG status (0-1) and absence of extra-cranial metastases were shown to be significantly associated with better survival outcomes after irradiation for CUP brain metastases [28], corroborating results from other studies examining brain metastases from various types of primary tumors and treatment modalities [9, 25, 27]. Most of these studies focus on whole brain radiotherapy (WBRT) with or without neurosurgical resection. Our results concur with those pertaining to the influence of ECOG status on survival for the CUP group. That is, lower ECOG status is statistically associated with improved survival outcomes. Similarly, age consistently inversely correlates with survival parameters [5], (Fig. 4).

The study is limited in a number of respects. First, precise data pertaining to the mode of surgical excision (en bloc versus piecemeal excision) extent of metastasis resection (gross total versus subtotal) was not recorded. Whilst differentiating the cohort further based on operative techniques and extent of resection would have been ideal this would have led to smaller cohort sizes precluding meaningful statistical comparisons. Similarly, sub-groups based on modality of radiation treatment were not created based on the same rationale. Second, this study is based on a small sample size, particularly the CUP cohort. Such a limitation is not unique to studying this patient population, particularly as neurosurgical treatment for patients with CUP metastases is relatively less common. This is one of the many drawbacks of retrospective studies as typically large sample sizes are necessary to examine for rare outcomes. In addition, our observations are limited by the inherent selection and information biases typical of retrospective studies. The evidence base concerning neurosurgical treatment of CUP metastases is not wide with differing methodologies; therefore, meaningful comparison of epidemiological data from this study with those undergoing similar treatment in other studies may be limited. Nevertheless, our study adds valuable insights to the evolving CUP brain metastases knowledge base.

These data reflect clinically relevant information gleaned during the transition to precision-era medicine, and before its latent effects are fully manifest. The National Research Council defines precision medicine as a means to tailor medical treatment to the individual qualities of individuals and the diseases of which they suffer. Furthermore, it is a means to stratify patients into subgroups differing in their responses to specific diseases as well as their varying responses to treatment [17]. Whilst the laudable goals of precision medicine are slowly becoming more achievable, particularly in oncological practice, in reality the unavailability of effective treatments for the many usually unfortunately means that precision medicine can rarely be practiced. The neurosurgeon and oncologist working today are still forced to act promptly to manage the often-deteriorating patient with CUP with a brain metastasis. In light of this, based on our data and others [12, 30], we would advocate the early consideration of metastasis excision surgery, even when a primary diagnosis remains undetermined.

## Conclusion

We have measured survival outcomes in a group of patients (CUP) who often present emergently to neurosurgical services. Comparison with a group of CKP patients demonstrates no significant difference in overall survival after neurosurgical intervention, suggesting surgery is a valid treatment option even before an underlying diagnosis is attained. The positive prognostic factors identified add to existing knowledge concerning the optimum treatments for patients with CUP brain metastases. Where prognostic factors indicate a poor survival outcome, less invasive and onerous treatment is perhaps more appropriate. We advocate large-scale prospective studies of this patient group to further refine the best treatment options.
